# Synthesis of Cross-Linked Chitosan and Application to Adsorption and Speciation of Se (VI) and Se (IV) in Environmental Water Samples by Inductively Coupled Plasma Optical Emission Spectrometry

**DOI:** 10.3390/ijms12064009

**Published:** 2011-06-14

**Authors:** Jun Dai, Feng Lian Ren, Chun Yuan Tao, Yang Bai

**Affiliations:** 1 College of Chemistry and Chemical Engineering, Central South University, Changsha, Hunan 410083, China; E-Mail: renfl2008@163.com; 2 College of Chemistry and Chemical Engineering, Jiujiang University, Jiujiang, Jiangxi 332005, China; E-Mails: taochunyuan@sina.com (C.Y.T.); boyang2618@163.com (Y.B.)

**Keywords:** cross-linked chitosan, speciation, inductively coupled plasma optical emission spectrometry, selenium

## Abstract

A new type of cross-linked chitosan was synthesized with Diethylene Triamine (DCCTS). The adsorption of Se (VI) on DCCTS was studied. The effect factors on adsorption and the adsorption mechanism were considered. The results indicated that the DCCTS could concentrate and separate Se (IV) at pH = 3.6; the maximum adsorption efficiency was 94%, the adsorption equilibrium time was 30 min; the maximum adsorption capacity was 42.7 mg/g; the adsorption fitted Langmuir equation. A novel method for speciation of Se (VI) and Se (IV) in environmental water samples has been developed using DCCTS as adsorbent and ICP–OES as determination means. The detection limit of this method was 12 ng/L, the relatively standard deviation was 4.5% and the recovery was 99%~104%.

## 1. Introduction

There has been an increasing demand for information regarding speciation of an element because the toxicity and biological activity of the element depends on its chemical form [1,2]. Selenium (Se) is one of the essential trace elements for humans. It is one composed element of red cell glutathione peroxidase [3]. Its main function is to compose peroxidase and protect cell membranes from damnifing [4]. Lack of selenium can cause several diseases for humans, such as big condyle disease andkeshan disease; whereas a high dose of selenium intake can be toxic [5–8]. The toxicity of selenium depends not only on its concentration but also its oxidation state. Different oxidation has different toxicity. The toxicity of different selenium species is: H_2_Se (−II) > Na_2_SeO_3_^2−^ (+IV) > Na_2_SeO_4_^2−^ (+VI) > Se (0). Se (0) has no toxicity [9]. The most prevalent oxidation states of selenium are Se (IV) and Se (VI) [10,11]. Speciation of Se (VI) and Se (IV) is necessary to evaluate the toxicological behavior of selenium.

Selenium is usually present at trace levels in environmental water samples, but its content in water is less than 10 μg/L [12]. Therefore, very sensitive techniques are necessary for determination of selenium in water samples such as atomic absorption spectrometry, atomic fluorescent spectrometry [13], inductively coupled plasma optical emission spectrometry (ICP–OES) [14], and inductively coupled plasma mass spectrometry [15]. Among these techniques, ICP–OES has desirable characteristics, such as high accuracy, high analytical efficiency, low detection limit, operational facilities and so on. However, the direct determination of selenium at very low concentrations is often difficult because of insufficient sensitivity as well as the matrix interferences occurring in real samples. For this reason, a preliminary preconcentration and separation step is often required. Selecting high efficiency concentration reagent shows its importance.

The methods used for preconcentration and separation include chemical precipitation [16], ion exchange [17], solvent extraction [18] and adsorption [19]. Among these methods, adsorption has been proved to be an efficient and economical technique. Activated carbon and silica gel are the two most popular adsorbents [20] in trace element analysis. However, they are relatively expensive materials since the higher the quality, the greater the cost. Looking for alternative adsorbents has intensified in recent years. At present, the focus is on chitosan (CTS). Chitosan is prepared from chitin by different degrees of deacetylation of the acetamido groups. Chitosan has both hydroxyl and amine groups that can be chemically modified [21–24], such as by cross-linking, ingrafting, alkylation, esterifing. Chemical modifications can offer a wide spectrum of tools to enhance the sorption properties of chitosan for metals. They may increase the chemical stability of the sorbent in acid media and, especially, decrease the solubility in most mineral and organic acids. They also increase its resistance to biochemical and microbiological degradation. A cross-linking step can reinforce the chemical stability of the biosorbents in such acid solutions. Although cross-linking reduces the adsorption capacity, it enhances the resistance of chitosan against acid, alkali and chemicals. Chitosan and its derivatives, have been reported to be used as adsorbents for metal ion [25], flocculant [26], carrier for medicine [27]. However, reports on using chitosan and its derivatives for preconcentration and separation of Se (IV) and Se (VI) anion in environmental water samples are rare.

In this work, we synthesized a new type of cross-linked chitosan with Diethylene Triamine, which has several amido groups. When chitosan was cross-linked with Diethylene Triamine, these amido groups were beneficial for DCCTS to sorb Se (VI) anion. Then using DCCTS as sorbent and ICP–OES as determination means, we studied the adsorption of Se (VI) on DCCTS, and formed a novel method for speciation of Se (VI) and Se (IV) in environmental water samples.

## 2. Results and Discussion

### 2.1. Characterization by Infrared (IR) Spectrum

**Figure 1:**
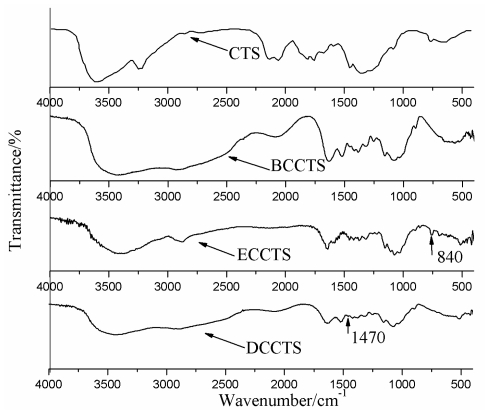
IR spectrum of Chitosan (CTS), Benzaldehyde condensating chitosan shiff alkali (BCCTS), Epichlorohydrine cross-linked chitosan intermediate (ECCTS) and Diethylene Triamine (DCCTS).

We analyzed the IR spectrum of the cross-linked product to validate whether the reaction performed according to our expectations to achieve the product. Figure 1 shows that Benzaldehyde condensating chitosan shiff alkali (BCCTS) presented flexural vibration adsorption peak of benzene ring at 758 cm^−1^, characteristic adsorption peak of benzene ring at 1580 cm^−1^ and flexural vibration adsorption peak of –C=N– at 1640 cm^−1^ [28]. This indicated that chitosan reacted with Benzaldehyde to obtain BCCTS. Figure 1 also showed that Epichlorohydrine cross-linked chitosan intermediate (ECCTS) presented vibration adsorption peak of epoxy group at 840 cm^−1^ besides the adsorption peak mentioned above. It did not present adsorption peak of C–Cl at 680–700 cm^−1^ [29]. This proved that halogenation reaction occurred between BCCTS and Epichlorohydrine to attain ECCTS. Figure 1(DCCTS) showed that DCCTS presented flexural vibration adsorption peak of –C–N–C– at 1470 cm^−1^ [30], whereas the epoxy group adsorption peak at 840 cm^−1^ disappeared. This confirmed that Diethylene Triamine succeeded to cross with ECCTS. Finally, the adsorption peak of benzene ring at 758 cm^−1^ and 1580 cm^−1^ disappeared, which showed that the protective group was depleted successfully in HCl solution.

### 2.2. Characterization by Scanning Electron Microscope (SEM) Image

**Figure 2:**
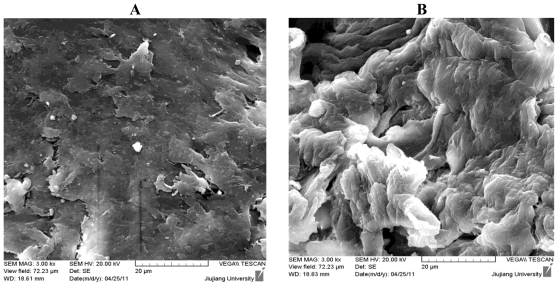
SEM images of (**A**) CTS and (**B**) DCCTS.

Figure 2 shows the SEM image of CTS and DCCTS. From the image, we could see that the surface of CTS was relatively smooth and the structure of CTS was compact. The surface of DCCTS was rough, with a lot of cavities on the surface and the structure of DCCTS was incompact. The reticular structure of DCCTS could enlarge its surface area (the surface area of DCCTS was 1.68 m^2^/g, whereas that of CTS was 1.45 m^2^/g) and enhance its adsorption ability.

### 2.3. Effect of pH on Adsorption of Se (VI) and Se (IV)

**Figure 3:**
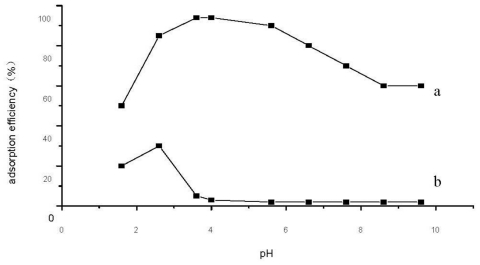
Effect of pH on the adsorption of (**a**) Se (VI) and (**b**) Se (IV).

A series of 2 μg/mL Se (IV) and Se (VI) standard solutions were prepared. Then their adsorption efficiency on DCCTS was determined under different pH according to Section 3.3. Figure 3 shows that DCCTS adsorbed Se (VI) strongly under acid conditions. The maximum adsorption efficiency of Se (VI) is 94% at pH 3.6, whereas 5% of Se (IV). DCCTS can concentrate and separate Se (VI) from Se (IV) solution at pH 3.6. This is because Se (VI) subsists in the solution mainly with SeO_4_^2−^ and HSeO_4_^−^ anions; however Se (IV) subsists with H_2_SeO_3_ molecule, which ionizes weakly [31].

Under acid conditions, amido groups on DCCTS react with H^+^, producing –NH_3_^+^. Thus DCCTS has a high positive charge, which adsorbs Se (VI) through electrostatic attraction. In a lower pH range (from 1.5 to 3.6), the adsorption efficiency increases with increasing pH value. This is because there are ionization balances in the solution as follows: H_2_SeO_4_ ↔ H^+^ + HSeO_4_^−^; HSeO_4_^−^ ↔ H^+^ + SeO_4_^2−^. When pH is lower, the concentration of H^+^ is higher; this would move the balance towards the left and decrease the amount of HSeO_4_^−^ and SeO_4_^2−^ anions. In other words, at this pH range (from 1.5 to 3.6), the higher the pH value the greater the amount of HSeO_4_^−^ and SeO_4_^2−^ anions. Therefore, the adsorption efficiency increases with the increasing pH value. At a higher pH range (from 3.6 to 9), although the ionization strengthens, the adsorption decreases due to decreasing protonated amido groups.

### 2.4. Adsorption Equilibrium Time and Adsorption Isotherm of Se (VI) on DCCTS

**Figure 4:**
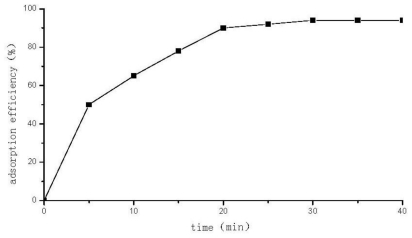
Adsorption equilibrium time of Se (VI) on DCCTS.

**Figure 5:**
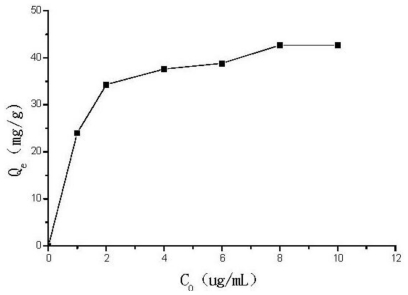
Adsorption isotherm of Se (VI) on DCCTS.

**Figure 6:**
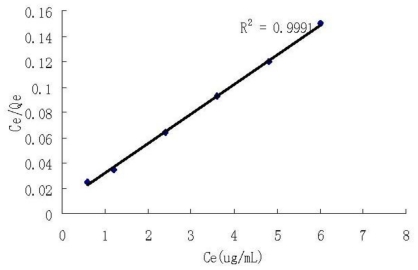
Fitting curve of adsorption by Langmuir equation.

At pH = 3.6, 2 μg/mL Se (VI) standard solution (200 mL) was prepared to decide the optimum adsorption equilibrium time. Figure 4 shows that under 30 min, the adsorption efficiency increases with increasing time, then at 30 min, the adsorption reaches an equilibrium; the adsorption equilibrium time of Se (VI) on DCCTS is therefore 30 min. Under room temperature, a series of different concentrations of Se (VI) standard solutions (200 mL) were then prepared. The pH of the solution was adjusted to 3.6. DCCTS (20 mg) was added into solution and the solution was filtered after 30 min. The concentration of Se (VI) was determined by ICP–OES. The adsorption capacity was calculated according to:

(1) $$Q_{e}=\frac{V(C_{0}-C_{e})}{W}$$

where *Q*_e_ (mg/g) is the adsorption capacity, *C*_0_ (μg/mL) is the initial concentration of Se (VI), *C*_e_ (μg/mL) is the equilibrium concentration of Se (VI), *V* (mL) is the volume of the solution of Se (VI), *W* (mg) is the weight of DCCTS added. Then we can get the adsorption isotherm by drawing the curve of *Q*_e_ against *C*_0_. Figure 5 shows that the maximum adsorption capacity of Se (VI) on DCCTS is 42.7 mg/g. Figure 6 is the fitting curve of the adsorption by Langmuir Equation:

(2) $$\frac{C_{e}}{Q_{e}}=\frac{C_{e}}{Q}+\frac{1}{Qb}$$

where *Q*_e_ (mg/g) is the adsorption capacity, *Q* (mg/g) is the maximum adsorption capacity, *C*_e_ (μg/mL) is the equilibrium concentration of Se (VI), *b* (mL/μg) is the Langmuir constant. From which we can deduce that the adsorption of Se (VI) on DCCTS is the Langmuir physics type of adsorption.

### 2.5. Mechanism of Adsorption of Se (VI) on DCCTS

Chitosan and its derivates adsorb metal cation mainly with chelation of amido groups [22,25,28,32], which belongs to chemical adsorption. However, this mechanism is not suitable for DCCTS adsorbing Se (VI) because Se (VI) exists in solution mainly with SeO_4_^2−^, HSeO_4_^−^ anion, which cannot be chelated by amido groups of DCCTS. As shown in Figure 6 and mentioned in Section 2.4, the adsorption of Se (VI) on DCCTS is the Langmuir physics type of adsorption. That is to say that the interaction between DCCTS and Se (VI) is a physical not a chemical interaction. As mentioned in Section 2.3, under acid conditions, amido groups on DCCTS react with H^+^, producing—NH_3_^+^; DCCTS has a high positive charge. On the other hand, SeO_4_^2−^ and HSeO_4_^−^ anions have a much more negative charge. Therefore, DCCTS can absorb Se (VI) through electrostatic attraction between the positive and negative charges [31]. This mechanism can be supported by the effect of pH on the adsorption of Se (VI) by DCCTS very well. Although at the narrow pH regions from 2 to 3, Se (IV) can be adsorbed by DCCTS, the adsorption efficiency is lower (as shown in Figure 3). The adsorption mechanism of Se (IV) on DCCTS is the same as Se (VI). As mentioned in Section 2.3, Se (IV) exists mainly with H_2_SeO_3_ molecule, which ionizes weakly. The electrostatic attraction between Se (IV) and DCCTS is so weak that Se (IV) cannot be well adsorbed by DCCTS.

### 2.6. Effects of Foreign Ions

The influences of some ordinary ions in the water were investigated. Various amounts of ions were added to a 2 μg/mL Se (VI) standard solution (50 mL) and the described procedure was followed. The results of this study are given in Table 1. From which we know that the major matrix ions show no obvious interference with the adsorption and determination of Se (VI), except Cr_2_O_7_^2−^. The interference of Cr_2_O_7_^2−^ can be avoided by adding diphenylcarbazide.

### 2.7. Characteristics and Application of the Proposed Method

Under optimum conditions, the blank solution determined ten replicates. The detection limit, based on three times the standard deviation of the blank, was 12 ng/L and the relative standard deviation was 4.5%. Table 2 compares the adsorption capacity of DCCTS used in this method with other adsorbents used in the control experiment or found in literature. From these sources, we can see that the adsorption capacity of DCCTS is the highest among these adsorbents.

In order to apply the proposed method, speciation of Se (VI) and Se (IV) in some environmental water samples (the pH value of water sample was adjusted to 3.6), including pond water, lake water, and tap water from Jiujiang University, China, were determined. At the same time, in order to validate the accuracy of the proposed method, different amounts of selenium were spiked in these environmental water samples. The results are given in Table 3. Good agreement was obtained between the added and the determined Se (VI) and Se (IV) types. The recovery values calculated for the standard additions were in the range of 99–104%. The proposed method could be applied successfully for the separation and speciation of trace amounts of selenium in environmental water samples.

## 3. Experimental

### 3.1. General

Chitosan (deacetylation degree 90%) was purchased from Shanghai National Reagent Company. The other reagents are all of analytical grade and offered by the chemistry laboratory of Jiujiang University. 0.1 mol L^−1^ HCl and 0.1 mol L^−1^ NaOH were used to control the pH values of the solutions. 1 g.L^−1^ Se (IV) and Se (VI) stock solutions were prepared by dissolving the appropriate amount of Na_2_SeO_3_ and Na_2_SeO_4_ in deionized water. Selenium was determined on an ICP–OES model Optima 5300 DV (PerkinElmer, USA). Its operating conditions are given in Table 4. pH values were measured on a pH meter model PHS-3C (Shanghai Precision Instrument Company, China). IR spectrum of the product was performed on an infrared spectrometer model Vertex70 (Bruker, Germany) with KBr disc method. The SEM image was performed on a SEM model Vega II (Tescan, Czech). The surface areas of the CTS and DCCTS were measured on a surface analyzer model ASAP 2010 (Micromeritics, USA) with the Brunauer-Emmett-Teller (BET) method.

### 3.2. Preparation of Diethylene Triamine Cross-Linked Chitosan

The process of preparation included the following steps:

#### (1) Synthesis of Benzaldehyde condensating chitosan shiff alkali

CTS (6.0 g) was dissolved in 1% aqueous solution of acetic acid (60 mL) for 30 min. Acohol (10 mL) was added to dilute the solution. Then adjusting the solution pH value to 5, Benzaldehyde (18 mL) was added to the solution with dropping funnel. The solution was stirred with heating at 70 °C for 4 h until the product was obtained. The solid product obtained was filtered off and washed several times with ethanol followed by distilled water. Then the product was dried at 60 °C in a vacuum drying oven.

#### (2) Synthesis of Epichlorohydrine cross-linked chitosan intermediate

The product obtained using Step (1) above was added to 0.4 mol L^−1^ NaOH aqueous solution (150 mL). The solution was heated at 55 °C. Epichlorohydrine (9 mL) was added to the solution with dropping funnel. The mixture was stirred for 5 h until the intermediate product was obtained. The product obtained was filtered off and washed several times with ethanol followed by distilled water. Then the product was dried at 60 °C in a vacuum drying oven.

#### (3) Synthesis of Diethylene Triamine cross-linked chitosan

The product obtained using Step (2) was added to 0.4 mol L^−1^ NaOH aqueous solution (150 mL).

Diethylene Triamine (15 mL) was added to the solution with dropping funnel. The solution was stirred with heating at 65 °C for 4 h until the product was obtained. The product obtained was filtered off and washed several times with ethanol followed by distilled water. Then the product was dried at 60 °C in a vacuum drying oven. The product was put into 1 mol L^−1^ HCl aqueous solution to get rid of Benzaldehyde. Thus the Diethylene Triamine cross-linked chitosan was prepared.

The schematic diagram of synthesis route referred to reference [32].

### 3.3. Adsorption, Desorption and Determination of Se (VI)

DCCTS (20 mg) was added to Se (VI) solution (200 mL) at 20 °C. Then the pH of the solution was adjusted to be 3.6 by 0.1mol L^−1^ HCl and 0.1 mol L^−1^ NaOH. The solution was filtered off after surging for 30 min. The DCCTS was washed several times with distilled water. 1 mol L^−1^ HCl (5 mL) was used to elute Se (VI) on the DCCTS. The final volume of eluting solution was 10 mL. The concentration of Se (VI) was determined by ICP–OES.

### 3.4. Determination of Total Se and Se (IV)

1% K_2_S_2_O_8_ aqueous solution was added to the water sample to oxidate Se (IV) to be Se (VI). Then the procedure was applied to determine total Se as described above. Se (IV) concentration was obtained as respective differences between total Se and Se (VI).

### 3.5. Control Experiment of Adsorption Capacity for Se (VI) on CTS, BCCTS, ECCTS and DCCTS

CTS, BCCTS, ECCTS and DCCTS (20 mg) was added to 8 μg/mL Se (VI) standard solution (200 mL) at 20 °C respectively. Then the procedure described in Section 3.3 was performed. The adsorption capacity was calculated by Equation (1) in Section 2.4.

## 4. Conclusions

In this work, a new type of cross-linked chitosan adsorbent (DCCTS) was synthesized with Diethylene Triamine. Compared to other sorbents, the DCCTS contained many amine groups, which could be protonated in acid solution. The protonated amido groups enhanced the ability of DCCTS to sorb Se (VI) anion in solution. This was the main advantage of using DCCTS as sorbent to preconcentrate and separate Se (VI) in water samples. The adsorption of Se (VI) on DCCTS was studied. The optimum conditions and the mechanism of adsorption of Se (VI) on DCCTS was discussed. Then using DCCTS as adsorbent and ICP–OES as determination method, a novel and useful speciation technique for Se (VI) and Se (IV) is offered. The presented procedure has been successfully applied for the separation and speciation of Se (VI) and Se (IV) in environmental water samples with acceptable accuracy and precision.

## Figures and Tables

**Table 1:** Influences of some foreign ions on the recoveries of Se (VI) (*n* = 3).

Ion	Added as	Concentration (μg/mL)	Recovery (%)
Na^+^	NaCl	2000	99.3
K^+^	KCl	2000	99.5
Mg^2+^	MgCl_2_	2000	98.8
Ca^2+^	CaCl_2_	600	98.2
Zn^2+^	ZnCl_2_	50	98.0
Cu^2+^	CuCl_2_	25	97.5
Fe^3+^	FeCl_3_	25	97.8
Cl^−^	NaCl	3000	96.5
NO_3_^−^	KNO_3_	3000	96.8
Cr_2_O_7_^2−^	K_2_Cr_2_O_7_	50	88.6

Data are expressed as mean of three replicates.

**Table 2:** Comparison of adsorption capacity for Se (VI) on DCCTS with other adsorbents.

Adsorbents	Adsorption Capacity (mg/g)	References
CTS	30.8	This work
BCCTS	28.6	This work
ECCTS	32.5	This work
DCCTS	42.7	This work
CCTS	34.5	[31]

**Table 3:** Speciation of Se (VI) and Se (IV) in environmental water samples (*n* = 3).

Water Samples	Se (VI) (μg/L)				Se (IV) (μg/L)			
	Found	Spiked	Recovered	Recovery (%)	Found	Spiked	Recovered	Recovery (%)
Pond water	0.710	0.20	0.916	103	0.580	0.20	0.784	102
Lake water	0.650	0.20	0.858	104	0.520	0.20	0.718	99
Tap water	0.420	0.20	0.622	101	0.250	0.20	0.452	101

Data are expressed as mean of three replicates.

**Table 4:** ICP–OES operating conditions.

ICP–OES Parameters
RF power 1300 W
Plasma gas (Ar) flow rate 15 L min^−1^
Carrier gas flow rate 0.24 L min^−1^
Sweeping rate 0.8 L min^−1^
Pumping rate 1.50 mL min^−1^
Analytical wavelength (Se) 203.9 nm
